# Remote Self-Administration of Cognitive Screeners for Older Adults Prior to a Primary Care Visit: Pilot Cross-Sectional Study of the Reliability and Usability of the MyCog Mobile Screening App

**DOI:** 10.2196/54299

**Published:** 2024-02-07

**Authors:** Stephanie Ruth Young, Elizabeth McManus Dworak, Greg Joseph Byrne, Callie Madison Jones, Lihua Yao, Julia Noelani Yoshino Benavente, Maria Varela Diaz, Laura Curtis, Richard Gershon, Michael Wolf, Cindy J Nowinski

**Affiliations:** 1 Department of Medical Social Sciences Feinberg School of Medicine Northwestern University Chicago, IL United States; 2 Center for Applied Health Research on Aging Feinberg School of Medicine Northwestern University Chicago, IL United States

**Keywords:** cognitive screening, cognitive, cognition, psychometric, usability, feasibility, early detection, dementia, Alzheimer’s disease, Alzheimer's, Alzheimer’s disease and age-related dementia, mHealth, mobile health apps, detection, screening, mobile health, mobile phone, app, apps, applications, applications, user experience, smartphone, smartphones, gerontology, geriatric, geriatrics, older adult, older adults, elder, elderly, older person, older people, ageing, aging, aged

## Abstract

**Background:**

Routine cognitive screening is essential in the early detection of dementia, but time constraints in primary care settings often limit clinicians’ ability to conduct screenings. MyCog Mobile is a newly developed cognitive screening system that patients can self-administer on their smartphones before a primary care visit, which can help save clinics’ time, encourage broader screening practices, and increase early detection of cognitive decline.

**Objective:**

The goal of this pilot study was to examine the feasibility, acceptability, and initial psychometric properties of MyCog Mobile. Research questions included (1) Can older adults complete MyCog Mobile remotely without staff support? (2) Are the internal consistency and test-retest reliability of the measures acceptable? and (3) How do participants rate the user experience of MyCog Mobile?

**Methods:**

A sample of adults aged 65 years and older (N=51) self-administered the MyCog Mobile measures remotely on their smartphones twice within a 2- to 3-week interval. The pilot version of MyCog Mobile includes 4 activities: MyFaces measures facial memory, MySorting measures executive functioning, MySequences measures working memory, and MyPictures measures episodic memory. After their first administration, participants also completed a modified version of the Simplified System Usability Scale (S-SUS) and 2 custom survey items.

**Results:**

All participants in the sample passed the practice items and completed each measure. Findings indicate that the Mobile Toolbox assessments measure the constructs well (internal consistency 0.73 to 0.91) and are stable over an approximately 2-week delay (test-retest reliability 0.61 to 0.71). Participants’ rating of the user experience (mean S-SUS score 73.17, SD 19.27) indicated that older adults found the usability of MyCog Mobile to be above average. On free-response feedback items, most participants provided positive feedback or no feedback at all, but some indicated a need for clarity in certain task instructions, concerns about participants’ abilities, desire to be able to contact a support person or use in-app technical support, and desire for additional practice items.

**Conclusions:**

Pilot evidence suggests that the MyCog Mobile cognitive screener can be reliably self-administered by older adults on their smartphones. Participants in our study generally provided positive feedback about the MyCog Mobile experience and rated the usability of the app highly. Based on participant feedback, we will conduct further usability research to improve support functionality, optimize task instructions and practice opportunities, and ensure that patients feel comfortable using MyCog Mobile. The next steps include a clinical validation study that compares MyCog Mobile to gold-standard assessments and tests the sensitivity and specificity of the measures for identifying dementia.

## Introduction

Primary care visits provide an important opportunity to detect pathological cognitive decline in the early stages [1,2], yet less than half of all cases are detected in primary care [3]. Medicare covers cognitive screening as part of the Annual Wellness Visit for adults aged 65 years or older, however, primary care clinics face several barriers to conducting regular cognitive screenings with their patients, including constraints on time and clinic staff [4]. Completing a screening remotely before a primary care visit offers several benefits to both patients and clinicians [5]. Patients can complete the screening at their leisure in the privacy of their own homes, and providers can review the results before a visit. Critically, a complete screener before a visit benefits all stakeholders (eg, clinicians, patients, and support staff) by saving time to address other important issues in person [6].

Mobile apps offer an ideal mechanism for many older adults to complete at-home cognitive screeners. More than 60% of older adults in the United States own a smartphone [7], and over 30% regularly use mobile health apps [8]. Moreover, low-income and minority groups are more likely to access their personal health information on smartphones compared to other electronic devices [9]. A small body of emerging research supports the feasibility of self-administered cognitive screeners on personal smartphones in research contexts [10-12]. The cognitive assessments in these studies vary in administration frequency, length, and structure but tend to find high levels of adherence (70% or higher), receive positive feedback in exit surveys, and show convergent validity with established cognitive screening measures [11,13,14]. However, no screeners to date have been validated for clinical use before a primary care visit [15]. Further research is needed to determine if older adults will be able to access the app and complete cognitive screeners independently, if the data collected from these screeners are reliable, and how older adults will perceive the app from a usability and acceptability perspective.

To encourage broader cognitive screening practices within primary care, the National Institute on Aging funded MyCog Mobile (1R01AG074245-01), a cognitive screening app that participants can self-administer remotely on personal smartphones and sends results directly to their primary care provider’s electronic health record. MyCog Mobile is the smartphone-based counterpart to MyCog, a tablet-based app that was developed for in-person self-administration in clinical settings [16]. MyCog Mobile uses 2 measures from MyCog adapted for remote assessment on a smartphone: Picture Sequence Memory (called MyPictures in the mobile app), which measures episodic memory, and Dimensional Change Card Sorting (called MySorting in the mobile app), which measures executive functioning. When combined with self-report, these 2 measures have demonstrated good sensitivity and specificity to detect cognitive impairment [16]. To expand the breadth of cognitive domains assessed, the pilot version of MyCog Mobile also includes 2 additional measures that are not in the original MyCog tablet app: a measure of working memory (MySequences) and a measure of memory for faces (MyFaces). We modeled each of the MyCog Mobile measures on existing mobile measures in the Mobile Toolbox [17], a comprehensive research platform and assessment library that allows for remote cognitive measurement on a personal smartphone (see *Measures* section).

MyCog Mobile is unique for 2 important reasons. First, it is a clinical screener meant to be used to help primary care providers make appropriate referrals and care recommendations, as opposed to a pure research measure such as the Mobile Toolbox. Second, MyCog Mobile is intended to be self-administered in a completely unsupervised remote setting, as opposed to the MyCog tablet app which is used in clinics under staff supervision. As such, MyCog Mobile underwent an extensive human-centered design process in which the platform and measures were optimized to be used by older adults in this context [5]. To ensure that the MyCog Mobile measures can be reliably self-administered by older adults in a remote setting, we piloted the screener in a sample of 51 adults aged 65 years or older who completed the measures on their personal iOS (Apple) smartphones. This pilot study will inform a subsequent construct and clinical validations, in which the sensitivity and specificity of the screener will be tested against clinical gold standards in a sample of healthy adults and used to differentiate healthy adults from those with cognitive impairment. Primary research questions for this pilot study include (1) Can older adults complete MyCog Mobile remotely on their smartphones? (2) Are the internal consistency and test-retest reliability of the measures acceptable? and (3) How do participants rate the user experience of MyCog Mobile?

## Methods

### Ethical Considerations

The research procedures were reviewed and approved by Northwestern University’s institutional review board (STU00214921). All participants provided informed consent and were compensated with a US $50 Visa gift card for their participation in this study. The data presented in this paper are anonymous and free of identifiers that could be linked to specific participants.

### Sample

We collaborated with a third-party market research agency to recruit older adults (N=51; Table 1) to take the measures on their smartphones twice, about 2 to 3 weeks apart. The agency contacted potential participants in their large database of thousands of older adults who had previously indicated interest in participation in research studies. Sample recruitment was broadly stratified by age, gender, racial and ethnic identity, and highest level of education. Inclusion criteria included (1) aged 65 years or older; (2) ownership of an iOS smartphone version 14 or higher; (3) being English-speaking; and (4) willing to complete the measures twice within approximately 2 to 3 weeks.

**Table 1:** Descriptive samples and sample demographics of pilot study participants.

Demographics			Total sample (N=51), n (%)
Age (years), mean (SD; range)			74.20 (6.25; 65-90)
**Gender**			
	Women	29 (57)	
	Men	22 (43)	
**Racial identity**			
	Black or African American	9 (18)	
	White	42 (82)	
**Ethnic identity**			
	Hispanic or Latino (any race)	5 (10)	
	Not Hispanic or Latino (any race)	46 (90)	
**Education level**			
	HS^a^ diploma or GED^b^	17 (33)	
	Some college	10 (20)	
	4-year college degree	12 (23)	
	Graduate or professional degree	12 (23)	

^a^HS: high school.

^b^GED: General Educational Development.

### Procedure

**Figure 1:**
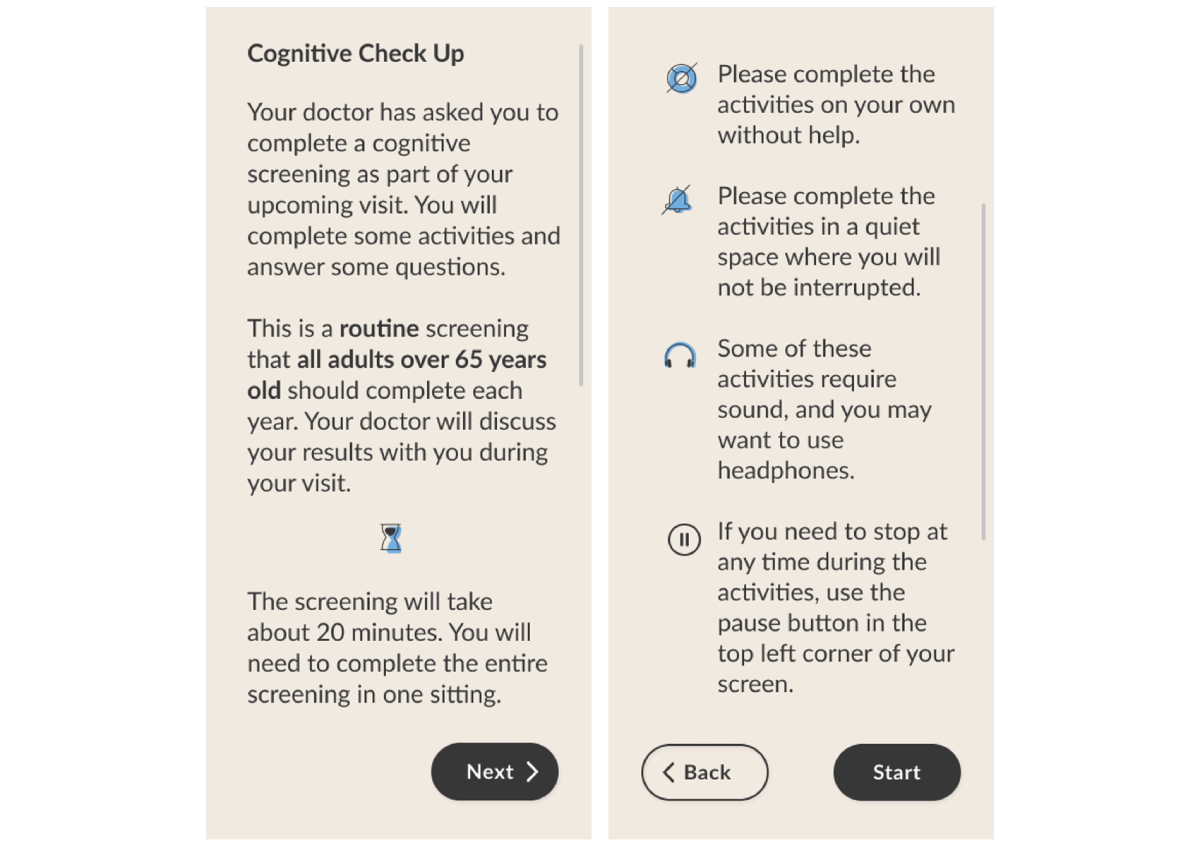
MyCog Mobile introduction screens.

Participants were asked to download the MyCog Mobile app onto their devices and complete the 4 activities in the battery and answer 2 demographic questions (age and education level). They received an email from this study’s staff with instructions to download the app and information on how to contact staff for support if needed. The app shows 2 brief intro screens (Figure 1) and then the cognitive screening begins with the learning trial of MyFaces. Participants then complete, in order, MySorting, MySequences, 2 demographics questions (age and education level), the recall subtests of MyFaces (see below), and, finally, MyPictures. After finishing their baseline MyCog Mobile assessment, participants completed a usability survey to provide feedback on their experiences. Participants were asked to self-administer MyCog Mobile a second time within 2 to 3 weeks of their baseline administration.

### Measures

#### MyFaces

**Figure 2:**
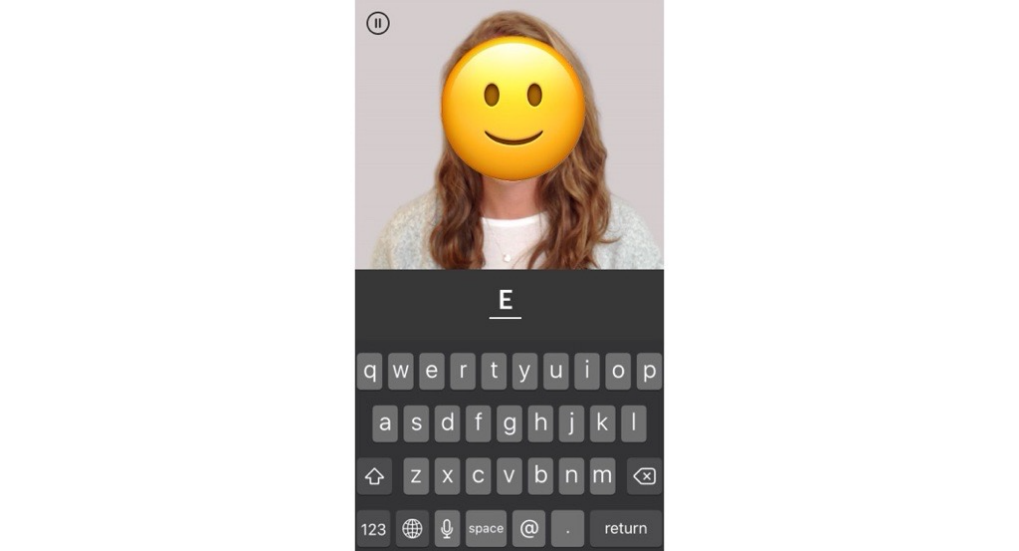
MyFaces first letter subtest example screen (face censored for publication).

MyFaces is an associative memory test originally developed by Rentz and colleagues [18] to predict cerebral amyloid beta burden. The MyCog Mobile version of this task was adapted from the Mobile Toolbox Faces and Names test, which was also based on the original test [17]. Participants are first shown 12 pictures of people paired with their names. After an approximately 5- to 10-minute delay, participants’ memories are tested in 3 subtests: the first subtest (recognition) asks the participant to select the person they saw in the learning trial from 3 options. The second subtest (first letter) asks participants to indicate the first letter of the name of the person presented on the screen (Figure 2). The third subtest (name matching) asks participants to select the name of the person presented from among 3 possible response options. A raw accuracy score is given for each of the 3 subtests.

#### MySorting

**Figure 3:**
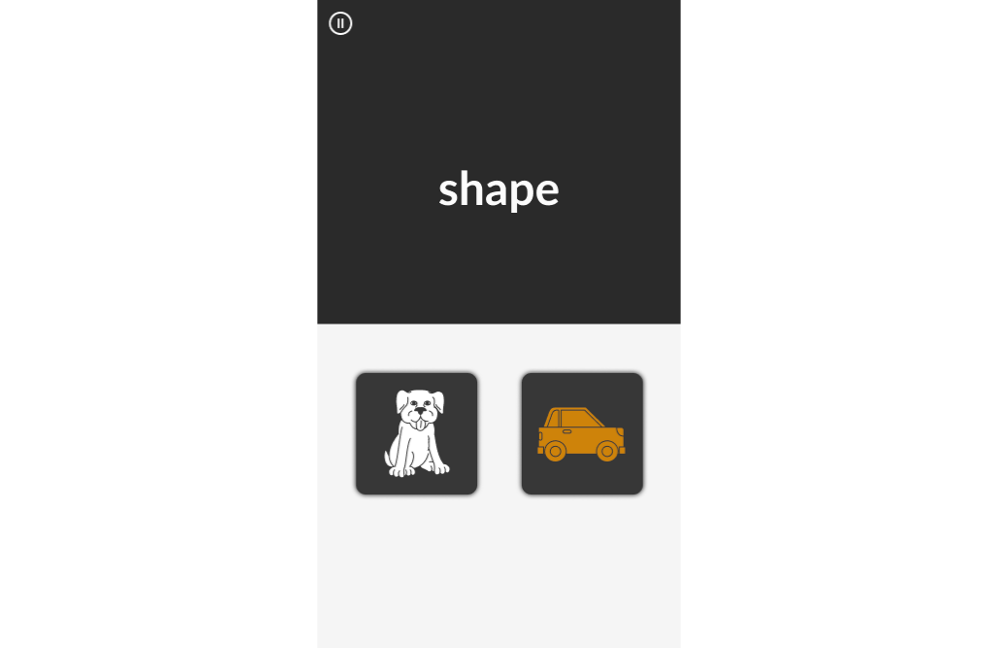
MySorting example screen.

MySorting is a measure of executive function and cognitive flexibility adapted from the MyCog Dimensional Change Card Sorting [16] and the Mobile Toolbox Shape-Color Sorting test [17]. Respondents are asked to sort images across 2 dimensions—shape and color—as quickly as they can. The relevant dimension for sorting is indicated by a cue word (“shape” or “color”) that appears on the screen (Figure 3). Scores are given for accuracy and response speed.

#### MySequences

**Figure 4:**
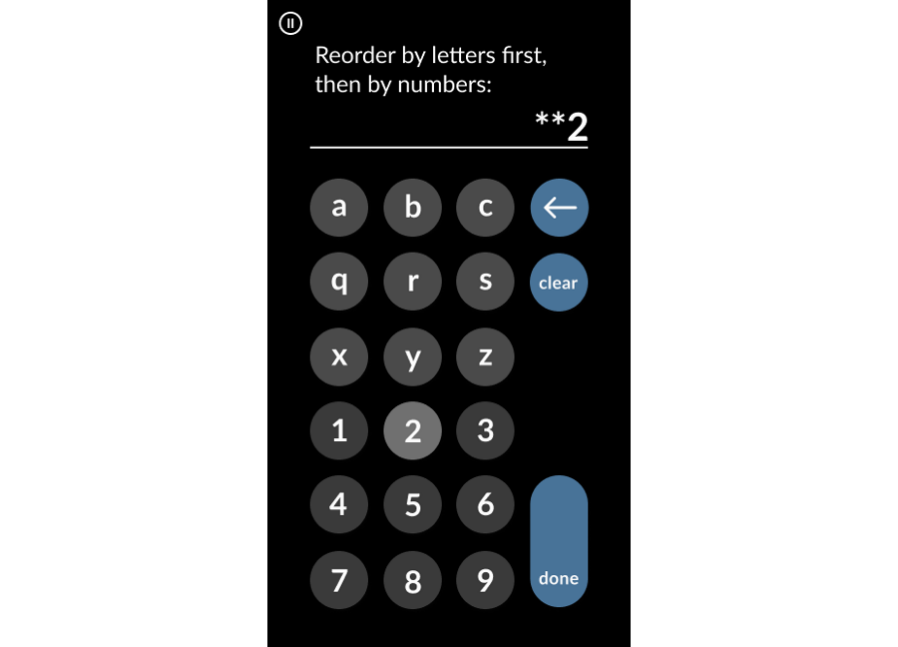
MySequences response entry example screen.

MySequences is a measure of working memory adapted from the Mobile Toolbox sequences test [17]. MySequences requires participants to remember strings of letters and numbers and arrange them in order, with the letters in alphabetical order first and then the numbers in ascending numerical order (Figure 4). Trials begin with strings of 3 alphanumeric characters and increase in length, reaching a maximum difficulty of 10 characters. Scores reflect the number of correct trials.

#### MyPictures

**Figure 5:**
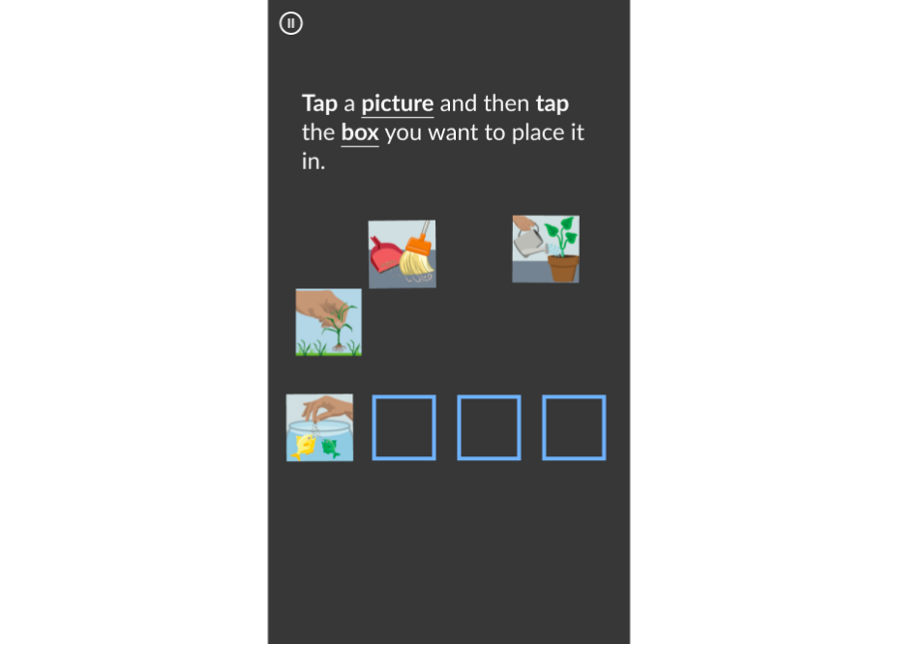
MyPictures practice example screen.

MyPictures is a measure of episodic memory adapted from the MyCog Picture Sequence Memory [16] and the arranging pictures task in the Mobile Toolbox [17]. A series of images depicting independent, nonsequential activities is presented in a specific order and placed in specific, sequential locations on the screen. Following this presentation, the images are scrambled, and the participant is asked to recall the original position of the images accordingly (Figure 5). There are 2 trials. Scores are given for exact match (the number of pictures in the correct positions) as well as adjacent pairs (the number of correctly ordered pairs of pictures next to each other) on each trial.

#### Simplified System Usability Scale

The Simplified System Usability Scale (S-SUS) is a modified version of the original System Usability Scale designed for adults aged 65 years and older with or without cognitive impairments [19]. Participants rate their level of agreement with statements about their experience using MyCog Mobile on a 5-point Likert scale. The original System Usability Scale has demonstrated evidence of its internal consistency, sensitivity to change, and concurrent validity with other usability measures.

#### Custom Usability Items

We also asked participants to respond to 2 additional 5-point Likert-scale items regarding their experience using MyCog Mobile: “the time to complete the MyCog Mobile Cognitive Screening was” (1=shorter than I expected, 3=about as much time as I expected, and 5=longer than I expected); and “how would you rate the experience of completing MyCog Mobile overall?” (1=very bad, 3=neutral, and 5=very good). Participants also provided feedback on the experience in 3 free-response items: (“what would you do if the app wasn’t working or you weren’t sure what to do next?”; “is there anything you would change about using the MyCog Mobile App to improve the experience?”; and “is there anything else you would like us to know about your experience using the MyCog Mobile app?”).

### Analysis

All analyses were conducted in R (R Core Team, 2023), and packages and codes are available on the Open Science Framework [20]. With 51 participants, we had 80% power to detect effect sizes of 0.38 or greater, which was adequate to evaluate our primary outcome of reliability metrics. Internal consistency was assessed using various methods that aligned with each task’s paradigm. For MySorting and MySequences, we calculated median Spearman-Brown correlations between bootstrapped random split-half coefficients for the accuracy scores. For MyPictures, we used the Pearson correlation between trial 1 and trial 2 adjacent pairs’ scores to calculate the Spearman-Brown split-half reliability (2*r*/(1+*r*)). For MyFaces, we used a look-up table to find expected a posteriori scores and SDs based on the sum of the accuracy scores across the 3 subtests [21,22] and then calculated the empirical and mean marginal reliabilities [23]. We considered internal consistency coefficients of 0.70 or greater to be acceptable [24]. We used intraclass correlations (ICCs) to evaluate test-retest reliability for each of the measures. ICCs and practice effects are reported for the MySorting total score, MySequences total score, MyPictures sum of adjacent pairs’ scores across trials 1 and 2, and the total score across all 3 subtests for MyFaces. We considered ICCs less than 0.50 to be poor, 0.50 to 0.75 acceptable, 0.75 to 0.90 good, and above 0.90 excellent [25]. Practice effects were evaluated through paired 2-tailed *t* tests of baseline and retest scores. CIs (95%) that contained 0 were considered to indicate nonsignificant practice effects.

We also conducted exploratory analyses of the relations between test performance, usability, and education, respectively, using Spearman ρ correlations. Spearman ρ correlations were used over Pearson *r* correlations because we were interested in monotonic relationships between variables rather than strictly linear ones. Correlations with age were not conducted due to the restriction of age range by study design. To assess the usability of the screener, we examined the score distributions on the S-SUS and custom Likert-scale items and qualitatively evaluated the results from custom usability survey items. A total score greater than 70 out of 100 possible points is considered above average and an acceptable level of usability [26,27]. Further, 2 authors independently reviewed and coded the free-response items. Codes were then reconciled, grouped, and categorized by representative themes. Although we counted each code’s frequency, the survey free-response items were an informal method of gathering feedback rather than a formal quantitative or qualitative study, and our analysis is exclusively descriptive.

## Results

### Overview

Most participants completed both administration time points within 15 days (mean_days_between_ 15.09 days, SD 2.08; range 13.12-22.38). Further, 2 participants did not complete the second MyCog Mobile assessment, leaving a sample of 49 participants for test-retest reliability analyses.

### Psychometric Properties

Internal consistency and test-retest reliability statistics were acceptable or better for each measure based on a priori cutoff criteria (Table 2). Test-retest reliability was moderate for each measure. Mean scores were not significantly different between baseline and retest except for MyFaces, which demonstrated a mean improvement of 4.70 (SD 1.06) in the total score across all 3 subtests at the second administration. The performance demonstrated moderate correlations with education level on each of the measures except MyPictures, which did not demonstrate significant correlations with education.

**Table 2:** MyCog Mobile measures reliability, practice effects, and correlation with education.

Measure	Internal consistency^a^ (95% CI)	Test-retest reliability (ICC^b^) (95% CI)	Practice effects (ΔM) (95% CI)	Education (ρ) (95% CI)
MyFaces	0.73 (0.63 to 0.82)	0.61 (0.40 to 0.76)	4.70 (2.62 to 6.79)	0.33 (0.05 to 0.56)
MySorting	0.90 (0.83 to 0.94)	0.71 (0.54 to 0.82)	1.75 (–0.97 to 4.49)	0.45 (0.19 to 0.66)
MySequences	0.91 (0.85 to 0.95)	0.65 (0.46 to 0.78)	1.77 (–0.43 to 3.96)	0.36 (0.08 to 0.58)
MyPictures	0.81 (0.73 to 0.94)	0.70 (0.53 to 0.82)	0.44 (–0.94 to 1.82)	0.06 (–0.22 to 0.33)

^a^Spearman-Brown corrected split-half correlations are reported for MySorting, MySequences, and MyPictures while empirical reliability is reported for MyFaces. Test-retest analyses are based on a sample of n=49.

^b^ICC: intraclass correlation.

### Usability

The mean overall usability rating on the S-SUS was acceptable (mean 73.17, SD 19.27). Ratings were not significantly correlated with education or performance on any of the measures. Analysis of the S-SUS items demonstrated Likert scale ratings in generally favorable directions (ie, positively worded items were greater than the neutral rating of 3, and negatively worded items were less than 3; Table 3). On additional custom Likert-scale items, participants indicated the time it took to complete the S-SUS was slightly less than expected on average, and the overall experience was positive.

**Table 3:** Usability ratings of MyCog Mobile.

Measure and item		Descriptive range	Rating, mean (SD)
**Simplified System Usability Scale**			
	I would use the MyCog Mobile app before an appointment with my doctor	Neutral to agree	3.53 (1.25)
	The MyCog mobile app is too complex for me	Disagree to strongly disagree	1.98 (1.16)
	The MyCog mobile app was easy to use	Agree to strongly agree	4.05 (0.97)
	I really need help from someone to use the MyCog mobile app	Disagree to strongly disagree	1.67 (0.105)
	The various parts of the MyCog mobile app were well integrated	Neutral to agree	3.78 (0.97)
	The MyCog Mobile app was confusing for me	Disagree to strongly disagree	1.98 (1.01)
	Learning to use the MyCog Mobile app was quick for me	Neutral to agree	3.75 (1.15)
	The MyCog mobile app was hard to use	Disagree to strongly disagree	1.90 (1.04)
	I felt confident using the MyCog mobile app	Neutral to agree	3.98 (1.12)
	I will need to learn a lot before using the MyCog mobile app	Neutral to disagree	2.04 (1.11)
**Additional Likert-scale questions**			
	The time to complete MyCog Mobile was...	As much time as expected or less	2.84 (1.07)
	How would you rate the experience of completing MyCog Mobile overall?	Good to very good	4.00 (0.94)

### Free-Response Feedback

On the first free-response item, “what would you do if the app wasn’t working, or you weren’t sure what to do next?” participants’ responses indicated several strategies they would use for help (Table 4). Most expected to be able to directly contact someone for support through an email or phone call. Several participants indicated they would use an in-app help resource or search for help resources online. Although most participants indicated they would try to solve the problem, 3 participants stated they would not finish MyCog Mobile if they encountered a difficulty (eg, “[I would] disregard it and continue on as I had before the app”).

On the second free-response item, “is there anything you would change about using the MyCog Mobile App to improve the experience?” most participants did not offer any suggestions or gave positive feedback (Table 4). Several participants suggested the instructions for the cognitive tests needed clarification (eg, “some of the exercises were not well explained or confusing. Particularly the ones where random letters and numbers were given, and they had to be reorganized. Simplifying the instructions would be helpful”). Some participants expressed concerns about their memories in response to this question or commented on the difficulties of the test items (eg, “I just wish I was smarter [and] had a better memory”). Regarding visual accessibility of the app, 2 participants indicated difficulty with the print size, and 1 indicated difficulty with the visual contrast of the tasks. Of note, 1 participant remarked they would prefer to complete MyCog Mobile in the clinic (eg, “while the app itself was easy & straight forward to download and access the survey material, I would most likely defer its home use and prefer an ‘in doctor’s office’ cognitive testing experience”). However, another participant remarked on how easy it would be to complete MyCog Mobile before the appointment (eg, “I really don’t see that anything was difficult. The app would work very well prior to a doctor visit.”). Further, 3 participants offered feedback on the process of participating in this pilot study, which will be considered for future study administration but is not relevant to the MyCog Mobile user experience specifically.

On the final free-response item, “is there anything else you would like us to know about your experience using the MyCog Mobile app?” most participants did not provide any feedback. As with the previous free-response item, several participants expressed concerns about their abilities (eg, “I found it to be quite challenging, especially since my memory isn’t what it once was.”). Some commented the instructions were confusing (eg, “no at 1st it was sort of confusing once I got into it, it was easier”), and 2 wanted more opportunities to practice before starting the live items. (eg, “I would like to see more practice questions to help the user feel more relaxed and confident”). Further, 2 participants wanted more explanation around the purpose of the test (eg, “perhaps a brief description of what the test is designed for, for example: to test mental recall, to test cognitive ability, to test onset of dementia or Alzheimer’s”). Only 1 participant reported difficulties loading the app for this study. Conversely, many participants provided positive feedback (eg, “it was simple and easy to use”).

**Table 4:** Free-response feedback on MyCog Mobile^a^.

Item and type of response			Frequency, n
“**What would you do if the app wasn’t working, or you weren’t sure what to do next?”**			
	Contact a support administrator	27	
	Self-troubleshoot or restart the app	13	
	Use in-app help or search online	7	
	Not finish or give up	3	
“**Is there anything you would change about using the MyCog mobile app to improve the experience?”**			
	No changes suggested or positive feedback	32	
	Clarify task instructions	7	
	Concerns about own abilities or test difficulty	5	
	Visibility issues	3	
	Concerns related to study administration	3	
	Preference for in-person experience	1	
“**Is there anything else you would like us to know about your experience using the MyCog mobile app?”**			
	No feedback	24	
	Positive feedback	14	
	Concerns about own abilities or test difficulty	5	
	Additional practice items	2	
	Purpose of test unclear	2	
	Difficulty loading app	1	

^a^Participant responses could be coded for multiple themes; therefore, the frequency should not sum to the total sample size.

## Discussion

### Principal Findings

The findings suggest most healthy older adults can reliably complete the MyCog Mobile screener remotely on their smartphones. The 4 performance measures that comprise the MyCog Mobile screener demonstrated acceptable internal consistency and test-retest reliability. The performance demonstrated positive correlations with education as expected, except for MyPictures, which did not correlate with education. Participants in our sample rated the usability of MyCog Mobile as above average and rated the experience “Good” to “Very Good” overall. They indicated the time to complete MyCog Mobile was about as long as they expected. These results provide evidence of the feasibility and acceptability of remote self-administration of the MyCog Mobile cognitive screener and support its further evaluation in larger clinical samples to understand its diagnostic accuracy and construct validity.

The feedback on free-response items indicated that most participants had a positive user experience and revealed several actionable insights for the next iteration of the MyCog Mobile app. First, participants expect a dedicated support representative to be available if they have difficulty using the app. Clinics that implement MyCog Mobile into their workflows will have to consider how to best respond to the needs of patients. For example, clinics may choose to dedicate resources for support or inform patients that the screener is optional, and they may defer the use of the app until their clinic visit if they encounter problems. Participants also indicated that they expect a help resource within the app. Currently, participants can use the “Pause” icon to stop the activities and review the instructions. However, an additional button labeled “Help” may be easier to navigate for older adults. Participants indicated that they would use several strategies to troubleshoot on their own if they encountered difficulties (eg, restarting the app); however, clinics should expect some participants not to finish MyCog Mobile if problems arise. For these patients, clinics will have to default to their previous screening workflows (eg, using in-person screeners like the MyCog tablet app or a traditional paper-and-pencil screener).

Concerning what could be improved with the app, some participants offered feedback on the instructions for the activities and asked for more opportunities to practice before completing live items. Although this feedback came from a minority of patients (7/51, 14%), we will conduct further cognitive interviewing to ensure instructions are optimized for all users. Currently, participants are only allowed to try practice items again if they respond incorrectly. However, adding the opportunity to try the practice again even if the item is correct may be helpful for participants. Further, 2 participants also reported trouble reading the print on a smartphone screen. To address this, we increased the font size of the print to maximize the readability of the text which will be implemented in the subsequent MyCog Mobile validation studies.

Several participants provided feedback that reflected insecurities about their abilities or performance on the test. Some reported the items were too difficult, however, the item difficulties cannot be changed to preserve the validity of the test. Instead of changing the items, steps could be taken to assure patients that it is normal for the items to be challenging. Based on feedback in a previous study, we designed the introduction screen (Figure 1) to alleviate potential concerns about the purpose of the test. For the next iteration of MyCog Mobile, we will collect participant feedback on how to optimize the introduction screen to make patients feel comfortable and assured when completing the screener at home.

### Limitations

The generalizability of our findings is limited by the relative homogeneity of our small sample about racial and ethnic identities. Representation of racial identities other than White was low or nonexistent, and representation of Hispanic or Latino populations was relatively small. Findings will need to be replicated in these populations in future studies to ensure MyCog Mobile has equal validity evidence for these groups. Moreover, due to the constraints of the grant, we developed the first version of MyCog Mobile for iOS devices (iPhones) only. iPhones are among the most expensive smartphones, which may have biased our sample toward higher-income participants (though we did not collect income information). Future work will focus on developing and validating MyCog Mobile for Android.

In this small pilot study, we were not able to conduct qualitative interviews with patients but rather gave them opportunities to provide feedback via free-response survey items. While free-response items can capture a breadth of spontaneous viewpoints, they may not achieve the depth or nuanced understanding of participants’ experiences and perspectives that can be gleaned from qualitative interviews. Consequently, our findings might not encompass the subtleties or the full range of participant experiences with MyCog Mobile.

The recruitment of older adults with cognitive impairments was outside of the scope of our pilot study; however, it is important to note that MyCog Mobile has yet to be researched in these populations. We expect older adults who are currently struggling with cognitive decline will likely have difficulty using MyCog Mobile, and the app may be more appropriate for participants who are cognitively intact or in the early stages of cognitive decline. The forthcoming clinical validation of MyCog Mobile will provide valuable information about the sensitivity and specify the measures to detect cognitive decline as well as the feasibility of using the app with cognitively impaired populations.

Further, 1 potential limitation of the MyCog Mobile app is older adults’ familiarity with mobile health apps in general. Although smartphone ownership is increasingly more common across age groups, some older adults still do not own smartphones or feel confident using them. The participants in our sample rated the user experience highly; however, the acceptability of a remote cognitive screening app is likely to be lower in a general population sample that has not chosen to participate in a highly controlled research study. Based on the results of the clinical validation, we will conduct a field test of MyCog Mobile, in which we will collect feedback on the acceptance of the app in real-world contexts.

### Comparisons With Prior Work

Our findings are consistent with the small body of research on the feasibility and acceptability of smartphone apps for cognitive screening but also offer some novel contributions. Several studies have examined repeated cognitive assessments on smartphones in research contexts for older adults, although these have primarily examined adherence [11,13,28]. Further, 1 ecological momentary assessment study found that both cognitively normal and older adults with mild cognitive impairment were able to complete cognitive assessments on their smartphones with an adherence rate of 85% in the context of a research study [29]. We observed a 96% adherence rate in our pilot study (albeit with only 2 administration time points), but it is unclear if patients will respond the same way when MyCog Mobile is used in the context of a real-world primary care visit, even if they are asked to complete the activities once annually.

Research on attitudes toward cognitive screening in primary care suggests that most older adults are open to cognitive screening in primary care if they perceive there is a benefit [30]. In our sample, the average response to “I would use the MyCog Mobile app before an appointment with my doctor” skewed positive, but 10 (20%) out of 51 participants responded with “Disagree” or “Strongly Disagree.” Future iterations of the app will focus on communicating the benefits of cognitive screenings to older adults, especially in the absence of clinical staff to explain the assessments when they are taken at home. Moreover, clinics should expect there to be a portion of patients who do not complete MyCog Mobile before the visit and will need to complete usual-care cognitive screenings in the clinic. MyCog Mobile is not intended to replace all in-person screening practices, but rather supplement such practices. Likewise, MyCog Mobile is not intended to provide a clinical diagnosis, but rather to identify potential cognitive impairment, and lead to appropriate referrals for more comprehensive evaluation. For the portion of patients who are willing and able to complete MyCog Mobile on their smartphones before their appointment, clinics can use in-person time to focus on other important aspects of the visit.

### Conclusions

Pilot evidence suggests the MyCog Mobile cognitive screener can be reliably self-administered by older adults on their smartphones. Participants in our study generally provided positive feedback about the MyCog Mobile experience and rated the usability of the app highly. Based on participant feedback, we will conduct further usability research to improve support functionality, optimize task instructions and practice opportunities, and make patients feel comfortable using MyCog Mobile. Additional next steps include a clinical validation study that compares MyCog Mobile to gold-standard assessments and tests the sensitivity and specificity of the measures for identifying cognitive impairment.

## Abbreviations

ICC: intraclass correlation
S-SUS: Simplified System Usability Scale

## Data Availability

Given the small sample size, data are not publicly available to ensure participants’ privacy. For questions about the data set, please contact the corresponding author.
